# The Non-Coding rs5945619 Variant at Xp11.22 and Its Putative Regulatory Effects on Nearby Genes in Ecuadorian Mestizo Women with Breast Cancer: A Case Series and In Silico Cis-eQTL Analysis

**DOI:** 10.3390/ijms27167085

**Published:** 2026-08-07

**Authors:** Rafael Tamayo-Trujillo, Ana Karina Zambrano, Patricia Guevara-Ramírez, Elius Paz-Cruz, Viviana A. Ruiz-Pozo, Santiago Cadena-Ullauri, Luis Israel Llerena Béjar

**Affiliations:** 1 Universidad UTE, Facultad de Ciencias de la Salud Eugenio Espejo, Centro de Investigación Genética y Genómica, Quito 170129, Ecuador; 2 Private Practice in Gynecology and Breast Health, Quito 170519, Ecuador

**Keywords:** breast cancer, rs5945619, Xp11.22, *LINC01496*, *NUDT11*, *GSPT2*, non-coding variants

## Abstract

Breast cancer (BC) is a heterogeneous disease influenced by genetic and regulatory mechanisms. Despite this, X-linked non-coding variants remain underexplored, particularly in admixed Latin American populations. Therefore, this study aimed to characterize the non-coding variant rs5945619 at Xp11.22 in Ecuadorian mestizo women with BC and to assess its potential regulatory effects on nearby genes through in silico cis-eQTL analysis. A prospective observational case series was conducted in 21 Ecuadorian mestizo women with histologically confirmed BC. Tumor DNA was extracted and analyzed using the Illumina TruSight Cancer Sequencing Panel. The rs5945619 variant was identified from sequencing data and compared with reference allele frequencies from dbSNP, ALFA, and the 1000 Genomes Project. Regulatory potential was assessed using RegulomeDB v2.2, and tissue-specific cis-eQTL associations were explored using GTEx. All 21 patients carried the genetic variant rs5945619 in a heterozygous state. The mean age at diagnosis was 54.5 ± 12.2 years, and invasive breast carcinoma of no special type was the predominant histological subtype. The T-allele frequency in the study cohort was 0.50, whereas Latin American reference populations showed higher frequencies ranging from 0.69 to 0.83. RegulomeDB assigned rs5945619 a rank of 1f and a functional score of 0.22, supporting its regulatory potential. GTEx analysis identified tissue-specific cis-eQTL signals, including *LINC01496* upregulation in testis, *NUDT11* and *LINC01496* downregulation in prostate, and modest *GSPT2* downregulation in mammary tissue. This study provides the first characterization of rs5945619 in Ecuadorian mestizo women with BC and suggests its role as a potential X-linked regulatory variant, requiring further validation. Although universal heterozygosity was observed, the small sample size, tumor-only design, and absence of a healthy control cohort prevent any causal, risk-factor, or tumor-driven selection inference. The in silico evidence supports a tissue-dependent regulatory model involving *LINC01496* and *NUDT11*, mainly in prostate/testis, and a modest *GSPT2* signal in mammary tissue. Therefore, breast cancer-specific eQTL analyses, functional validation, and larger ancestry-informed case–control studies are required to determine the biological and clinical relevance of this locus.

## 1. Introduction

Breast cancer (BC) is one of the most commonly diagnosed malignancies and remains a leading cause of cancer-related mortality, including among women of reproductive age [1,2]. Its increasing incidence, socioeconomic burden, and high molecular and clinical heterogeneity further aggravate prevention, diagnosis, and treatment strategies [1,2]. Accordingly, ongoing research is primarily focused on identifying diagnostic biomarkers, prognostic indicators, and therapeutic targets that may improve disease management.

Inherited susceptibility to BC is partly attributable to pathogenic germline mutations in high- and moderate-penetrance genes, including *BRCA1/2*, *PALB2*, *ATM*, *CHEK2*, *RAD51C*, *BARD1*, and *TP53* [3]. However, inherited pathogenic variants account for only an estimated 5–10% of BC cases, and disease risk is also influenced by hormonal, environmental, and lifestyle factors [2,3]. Whole-Genome Sequencing (WGS) has expanded the investigation of BC susceptibility beyond established coding genes by identifying additional candidate loci and pathways, including those involved in estrogen biosynthesis and alternative DNA repair mechanisms [2,4,5]. WGS has also allowed the description of historically overlooked genomic elements like non-coding regulatory regions, including long intergenic non-coding RNAs (lincRNAs). These transcripts can interact with DNA, RNA, and proteins and may regulate chromatin organization, stability, and translation in tissue-specific signaling pathways [6,7,8]. Consequently, the detection of variants affecting lincRNA loci and their regulatory activity is being investigated as potential biomarkers and therapeutic targets [6].

Several studies have described the role of rs5945619 in cancer development, primarily in prostate cancer susceptibility [9,10,11,12]. This X-linked variant is located at position 51,498,820 within a non-coding region of the X chromosome, and it involves a cytosine-to-adenine or cytosine-to-thymine substitution [13]. This genetic variant has been strongly associated with prostate cancer risk, as it was found to be primarily transmitted to affected offspring in families with a history of prostate cancer [12]. Furthermore, the approximately 140 kb region encompassing this variant at Xp11.22 has been associated with prostate cancer, potentially through regulatory effects on *LINC01496* (long intergenic non-protein coding RNA 1496) and the neighboring *NUDT10* and *NUDT11* genes [12,14].

*NUDT10* and *NUDT11* are involved in nucleotide metabolism and cellular processes like apoptosis, endocytosis, telomere length maintenance, gene repair, and chemotaxis [15,16]. Their potential involvement in cancer pathogenesis has encouraged research into their utility as therapeutic targets and as prognostic or diagnostic biomarkers [17,18]. These genes are located within a region of extended linkage disequilibrium on the X chromosome, which may also influence the development of BC in Ecuadorian mestizo women [12,14].

The X chromosome could also contribute to BC through its genetic and epigenetic regulation. In female embryonic cells, one of the two X chromosomes is randomly inactivated, although ~23% of X-linked genes may escape X-chromosome inactivation [19,20]. Disruption or loss of X-chromosome inactivation has been associated with altered expression of X-linked genes and with tumor-related phenotypes, including aggressive characteristics in some BC models [21,22]. Despite these characteristics, the X chromosome remains underrepresented in genome-wide association studies (GWASs), limiting the understanding of its contribution to BC susceptibility and sex-related differences in cancer risk [23,24]. This limitation is highly relevant in admixed populations, including Ecuadorian populations, which remain insufficiently represented in genomic studies.

Therefore, this study aimed to describe the rs5945619 genetic variant in Ecuadorian mestizo women with BC and to explore its potential regulatory role within the Xp11.22 region. We assessed whether this variant shows in silico cis-eQTL associations with nearby genes (*NUDT10* and *NUDT11*) and *LINC01496*, which may contribute to BC oncogenic mechanisms, while avoiding inference of BC causality or clinical actionability.

## 2. Results

### 2.1. Clinical Characteristics of Cases

Twenty-one Ecuadorian mestizo women with histopathology-confirmed BC were enrolled. The mean age at diagnosis was 54.5 ± 12.2 years (range: 34–81 years). The predominant histological type was invasive breast carcinoma of no special type (IBC-NST) (*n* = 17, 80.96%), followed by invasive lobular (*n* = 2, 9.52%), invasive mucinous carcinoma (*n* = 1, 4.76%), and invasive breast carcinoma of no special type with a micropapillary component (*n* = 1, 4.76%). Immunohistochemical subtyping classified 11 cases as luminal A (52.3%), three as luminal B (14.3%), three as HER2-enriched (14.3%), three as triple-negative breast cancer (TNBC) (14.3%), and one type without information (4.8%). Clinical and pathological characteristics are summarized in Table 1.

### 2.2. Detection of rs5945619

Next-Generation Sequencing was performed for all 21 samples, and rs5945619 was identified in every case. All individuals harbored the variant allele in a heterozygous state (C/T), carrying one wild-type cytosine allele and one variant thymine allele. The variant maps to chromosome X:51,498,820, positioned within a non-coding regulatory region near *NUDT10*, *NUDT11* and *LINC01496* at Xp11.22, and represents a C-to-T single-nucleotide substitution.

Reference allele frequency data retrieved from the Allele Frequency Aggregator (ALFA) indicate that the T allele is common and, in several Latin American subgroups, constitutes the major allele: frequencies of 0.69 and 0.83 were reported for Latin American 1 and Latin American 2 cohorts, respectively; the 1000 Genomes American panel recorded a T-allele frequency of 0.82. These results underscore that, in the reference populations, most genetically proximate to our cohort, the T allele is not rare. Population-stratified allele frequency data are presented in Table 1.

### 2.3. Predicted Regulatory Effects

RegulomeDB v2.2 assigned rs5945619 a categorical rank of 1f, denoting strong evidence for regulatory activity of the variant, supported by data from quantitative trait locus and transcription factors, but lacks an experimentally verified motif match. The functional probability score was 0.22, suggesting that the variant may influence gene regulation in at least one tissue context. However, the supporting evidence was concentrated mainly in androgenic activity tissues (testis and prostate), rather than mammary tissue.

**Figure 1:**
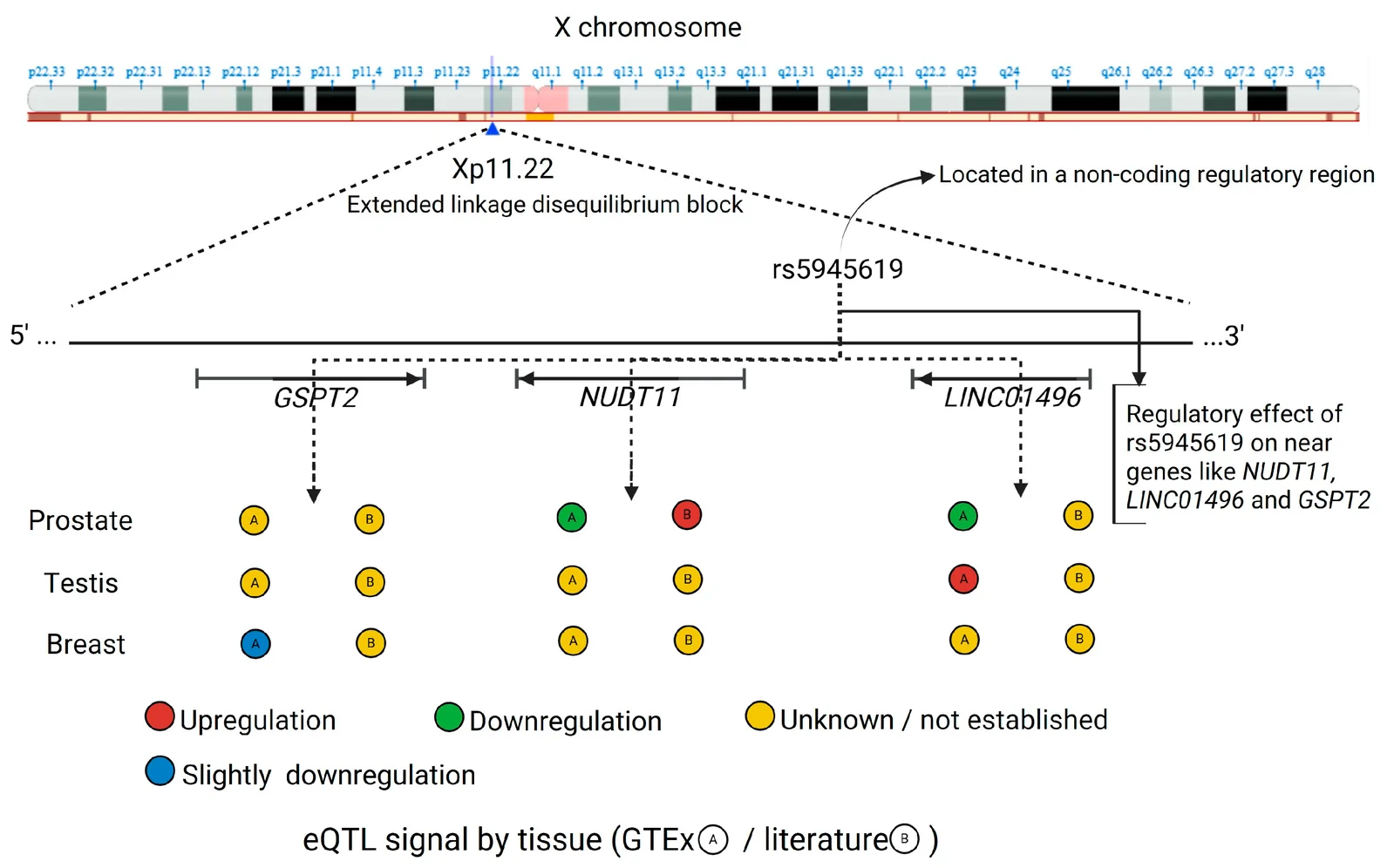
Schematic representation of rs5945619 within an extended linkage disequilibrium block at Xp11.22. The variant is located in a non-coding regulatory region that could influence nearby genes, including *GSPT2*, *NUDT11*, and *LINC01496*. Tissue-specific eQTL signals in prostate, testis, and breast tissue are shown based on GTEx data and the published literature. *NUDT11* upregulation in prostate tissue has been previously reported in prostate cancer; eQTL: expression quantitative trait locus.

The GTEx v8 single-tissue eQTL database identified tissue-specific cis-eQTL associations for rs5945619. In testis, the T allele was associated with *LINC01496* upregulation (normalized effect size [NES] = +0.41; *p*-value = 2.6 × 10^−53^). In prostate tissue, the same allele was associated with *NUDT11* downregulation (NES = −0.44; *p*-value = 1.5 × 10^−36^) and *LINC01496* downregulation (NES = −0.42; *p*-value = 4.4 × 10^−19^). In mammary tissue, rs5945619 showed only a modest association with *GSPT2* downregulation (NES = −0.084; *p*-value = 0.000069) (Figure 1). Therefore, the eQTL evidence should be interpreted as tissue-dependent, and the current data do not establish a breast cancer-specific regulatory mechanism for *NUDT11* or *LINC01496*. The regulatory annotation data are summarized in Table 2.

## 3. Discussion

The present case series documents, for the first time, the genotypic distribution of rs5945619 in Ecuadorian mestizo women with BC previously characterized at the histopathological and genomic level [25]. All 21 cases carried the variant in a heterozygous state, with no homozygous wild-type or homozygous variant genotypes detected. Because the study lacks a healthy Ecuadorian control cohort and includes a small number of tumor-only samples, this finding should be interpreted as a preliminary observation rather than as evidence of disease association, tumor-driven selection, or BC risk. The in silico analysis placed rs5945619 within a putative regulatory region at XP11.22 and identified tissue-dependent cis-eQTL signals involving *LINC01496*, *NUDT11*, and, more modestly, *GSPT2*.

The SNP rs5945619 is located within a non-coding regulatory region at Xp11.22 that has been associated with increased susceptibility to prostate cancer, particularly in familial and early-onset cases [26]. This locus lies within a genomic block characterized by extended linkage disequilibrium, suggesting that rs5945619 may act as a regulatory element of neighboring genes [27]. This region encompasses *LINC01496* and *NUDT11*, which showed a regulatory effect of this SNP in an in silico analysis performed in this study. Previous functional and eQTL studies have shown that risk alleles at rs5945619 are associated with overexpression of *NUDT11* in prostate cancer tissue [18,28]. The eQTL analysis performed in this study showed a downregulation of the *NUDT11* gene; however, this was performed in normal tissues, which could explain the contradictory effects of rs5945619 on *NUDT11* gene expression in prostate cancer tissue (Figure 1). The *NUDT11* gene is involved in nucleotide metabolism and cellular signaling processes, whose overregulation may contribute to tumorigenesis via altered cellular homeostasis and proliferation [12,14].

The regulatory potential of this non-coding regulatory region could also be mediated by chromatin interaction and enhancer activity, which may reinforce the hypothesis that non-coding variants can influence cancer risk via transcriptional modulation of target genes [6,14,26]. Moreover, the variant detected here may be part of enhancer, promoter, or silencer elements, non-coding RNAs, or microRNAs, which could regulate gene expression at both transcriptional and post-transcriptional levels, influencing critical cellular processes such as proliferation, differentiation, and apoptosis [7,8,29]. This approach must be assessed in future studies integrating regulatory annotation, chromatin accessibility assays, histone modification profiling, eQTL analysis, reporter gene assays, and CRISPR-based functional validation. These findings highlight the importance of considering regulatory mechanisms by non-coding genomic elements in the genetic architecture of cancer susceptibility.

In the 1000 Genomes Admixed American and ALFA Latin American subgroups, the rs5945619 T allele has frequencies between 0.69 and 0.83, indicating it is a common allele in these populations. Under HWE applied to a diploid female population, a T-allele frequency of approximately 0.83 would predict heterozygous genotype frequencies of roughly 28%. Thus, the absence of both reference-homozygous and alternate-homozygous genotypes would be unexpected under random sampling from a population in HWE [30]. However, because the present study included a small, tumor-only case series without a population-based control group, the observed heterozygosity cannot be interpreted as evidence of tumor-driven selection. It should instead be regarded as a hypothesis-generating observation requiring validation in larger, ancestry-stratified case–control studies using germline DNA. Population admixture and ancestry-specific linkage disequilibrium patterns may also influence the distribution of rs5945619 genotypes in Ecuadorian mestizo populations [31].

Prostate cancer is a genetically, phenotypically and clinically heterogenous disease that originates mainly from prostate epithelial cells and is driven by mutations and amplification of androgen receptor signaling, gene fusions of the E26 transformation-specific family (*ERG*, *ETV*, *ETV4*, *FLI1*) with the androgen-regulated gene *TMPRSS2*, mutations in *SPOP*, *FOXA1*, *TP53*, *BRCA2*, *PTEN* and *RB1*, activation of PI3K, WNT/β-catenin, SRC, IL-6/STAT3 and MAPK signaling pathways, and inactivation of DNA repair genes [32]. These alterations contribute to tumor initiation, progression, cell-lineage plasticity, and treatment resistance [32,33]. Moreover, the genetic landscape of this disease is expanding due to GWAS that identified new prostate cancer risk loci, like the SNP rs5945619 in the Xp11.22 region [26]. The prostate cancer risk is increased in men of African American descent [34]; however, rs5945619 has been detected with the same magnitude in men of European and African descent [35]. Moreover, GWASs may also identify risk factors for other malignancies, such as the risk of pancreatic cancer in men with Lynch Syndrome [36] or pancreatic metastasis after primary intrahepatic cholangiocarcinoma [37], since Lynch Syndrome and cholangiocarcinoma have been described in our population [38,39].

The regulatory annotation data from RegulomeDB and GTEx supported a tissue-dependent regulatory model. The strongest cis-eQTL signals were detected in androgen-responsive tissues (prostate and testis) for *LINC01496* and *NUDT11*. Moreover, a weak eQTL signal in breast tissue for *GSPT2* was detected, while no signal was found for *LINC01496* or *NUDT11* in this tissue. This finding could indicate that rs5945619 within the Xp11.22 regulatory block may exert tissue-specific regulatory effects on nearby genes (Figure 1). Moreover, tumor-specific eQTL studies have shown that variants with no eQTL signal in GTEx normal tissue exert measurable expression effects within the tumor microenvironment [40]. Hence, these results indicate that *GSPT2* is the most directly supported breast-tissue candidate for regulation by rs5945619 in this in silico analysis.

However, since GTEx mammary tissue samples are predominantly from post-menopausal women rather than cancer tissue, breast cancer-specific eQTL studies and functional assays are required before assigning a mechanistic role to this locus in BC. Therefore, these prostate-specific findings should not be extrapolated directly to BC pathogenesis, since in the present analysis, they are used only to contextualize the regulatory background of the locus and to justify further investigation of X-linked non-coding variation in admixed Latin American populations.

*GSPT2* is also located at Xp11.22 and encodes eukaryotic peptide chain release factor 3b (eRF3b), a GTPase involved in the conformational change of eukaryotic release factor 1 (eRF1) by GTP hydrolysis, which triggers translation termination by dissociation of proteins from the ribosome [41]. This function links *GSPT2* to post-transcriptional control and translation processes that may be altered during tumorigenesis. Although direct evidence connecting *GSPT2* with BC remains limited, its biological role provides a plausible mechanistic framework through which regulatory variation at Xp11.22 could affect cancer-relevant pathways beyond nucleotide metabolism. The modest breast-tissue eQTL signal observed for *GSPT2* suggests that this gene could be considered an additional candidate target of the rs5945619-containing regulatory region (Table 2) that requires validation in breast normal tissue and breast tumor models. Moreover, functional studies are required to determine whether this regulatory association has relevance in BC pathogenesis, diagnosis, and treatment strategies.

The evidence of *NUDT11* expression in BC tissue is limited; however, the Human Protein Atlas (HPA) shows expression of this protein in normal and oncogenic breast tissue but does not identify *NUDT11* gene expression as prognostic in breast invasive carcinoma [42,43,44]. The lack of functional analysis of this gene could be due to the limited inclusion of the X chromosome in BC GWAS. Functional validation studies of breast cancer-specific eQTL and histopathological assays could demonstrate the presence or absence of *NUDT11* expression in BC tissues and the role of rs5945619 in *NUDT11* regulation. As described previously, there is a clear expression of the *NUDT11* gene in androgenic tissues like prostate and testis, and alterations in androgen receptor signaling could act as triggers of prostate carcinogenesis [32,33]. Thus, *NUDT11* should be considered a candidate gene supported mainly by prostate/testis regulatory evidence and by its location within the Xp11.22 linkage disequilibrium block, rather than as a demonstrated BC effector.

Moreover, there is evidence that androgen signaling is involved in BC pathogenesis and its molecular characterization in breast tissue could be used for the development of personalized medicine strategies [45,46]. The current dataset does not support a direct regulatory relationship between rs5945619 at Xp11.22 and the androgen receptor (*AR*) locus at Xq12; however, since rs5945619 is located in an extended linkage disequilibrium block of a non-coding regulatory region on the X chromosome, this variant could influence the expression of distant genes like the androgen receptor gene, which could overexpress and overactivate androgen signaling at the breast tissue level. This hypothesis remains biologically tenuous without supporting chromatin conformation data (e.g., Hi-C or ChIA-PET) and should be regarded as speculative pending such evidence [47,48]. This hypothesis must be proved in standardized protocols that could contribute to BC biology.

Although a direct association between rs5945619 and BC has not yet been established, its location in an extended linkage-disequilibrium region of the X chromosome and its potential regulatory effect on *GSPT2*, *NUDT11* and *LINC01496* provide a biological field for its investigation in BC pathogenesis, particularly in admixed populations. Since X-linked regulatory variants may be influenced by X-chromosome inactivation and ancestry-specific linkage disequilibrium patterns, rs5945619 could potentially affect *GSPT2*, *NUDT10, NUDT11* and *LINC01496* expression in breast tissue and contribute to tumor-related processes such as impairments in nucleotide metabolism, DNA repair processes, and cellular stress responses.

### Study Limitations and Future Perspectives

The present findings should be interpreted within the limitations of a small case series without healthy controls. However, the present study establishes a starting point for future investigations. The most immediate scientific opportunity is to determine whether rs5945619 heterozygosity is a universal finding across both healthy and affected populations, or whether it is specifically enriched among men with prostate cancer or women with BC. To address this, a population-based case–control study with a substantially higher number of subjects is needed, designed to estimate the frequency of the variant allele across different population groups, including admixed Latin American cohorts, and to compare genotype distribution between cases and controls with statistical power and ancestry-stratified correction.

The transition from a regulatory prediction to a demonstrated molecular mechanism remains a critical next step. Functional genomic experiments designed to compare the transcriptional activity conferred by the wild-type and the variant alleles are required, particularly in specific tissue models.

The two loci for which rs5945619 acts as a cis-eQTL, *LINC01496* and *NUDT11*, belong to distinct functional categories. *LINC01496* is a long intergenic non-coding RNA implicated in transcriptional regulation, while *NUDT11* encodes a protein involved in nucleotide metabolism and signaling. Characterizing the expression pattern of both loci across multiple tissue types will be essential for understanding the full scope of their contribution to carcinogenesis.

Moreover, multi-omics integration combining genotyping data with RNA-seq, proteomics, and metabolomics in a larger Latin American patient cohort would further allow comprehensive characterization of the downstream metabolic consequences of rs5945619 heterozygosity. This approach could capture the propagation to the protein layer and to the small-molecule metabolite landscape and provide a system-level view of tumor biology.

A regional research framework including Ecuadorian and broader Latin American cohorts would be valuable for improving ancestry-informed interpretation of X-linked non-coding variants. At present, these findings do not support clinical actionability or precision oncology applications, but they provide a basis for future functional and population-based studies.

## 4. Materials and Methods

### 4.1. Study Design and Population

A prospective observational case series was conducted, including women diagnosed with histopathology-confirmed BC in Quito, Ecuador. The case series design was chosen as an appropriate and ethically efficient framework for characterizing a previously undescribed genotype-disease relationship in a population that remains substantially underrepresented in molecular epidemiological research. Cases were enrolled consecutively, and inclusion was restricted to women who self-identified as Ecuadorian mestizo born in Ecuador across at least three generations. All participants provided written informed consent prior to any study procedure, and the protocol was approved by the Institutional Ethics Review Board.

Twenty-one women met eligibility criteria and were enrolled. Clinical and histopathological data, including age at diagnosis, tumor histological type, and immunohistochemical (IHC) receptor profile, were provided by the physician.

### 4.2. Sampling and DNA Extraction

Tumor samples were collected from biopsy specimens and transferred to 0.9% sodium chloride solution for preservation. Genomic DNA was isolated using the PureLink Genomic DNA Mini Kit (Thermo Fisher Scientific, Waltham, MA, USA), strictly following the manufacturer’s protocol. DNA yield was assessed fluorometrically with the Qubit Broad Range dsDNA Assay Kit (Thermo Fisher Scientific, Waltham, MA, USA). All samples were normalized to a working concentration of 5 ng/µL prior to library preparation.

### 4.3. Targeted Next-Generation Sequencing

Targeted next-generation sequencing was performed using the TruSight Cancer Sequencing Panel (Illumina, San Diego, CA, USA). Library preparation and sequencing were carried out according to the manufacturer’s instructions, with sequencing performed on the MiSeq System (Illumina, San Diego, CA, USA) at the Centro de Investigación Genética y Genómica, Universidad UTE, Quito. Read alignment was performed using the DRAGEN Enrichment pipeline (v3.10.4) against the GRCh38/hg38 human reference genome, followed by joint variant calling. The resulting variant call files were used for all downstream annotation and functional analyses.

### 4.4. Data Analysis

Variant rs5945619 was cross-referenced against the NCBI Single Nucleotide Polymorphism Database (dbSNP, build 156) and the Allele Frequency Aggregator (ALFA) to retrieve population-level allele frequencies across continental and subcontinental reference groups, including the 1000 Genomes Project Phase 3 Admixed American (AMR) cohort [13].

Regulatory potential of rs5945619 was characterized in silico using RegulomeDB version 2.2 (https://regulomedb.org/), which integrates data from ENCODE, Roadmap Epigenomics, and GTEx to assign functional scores to non-coding variants [49].

Expression quantitative trait locus (eQTL) analysis was conducted using the GTEx portal, querying all available single-tissue cis-eQTL associations for rs5945619 across the full GTEx tissue compendium (https://gtexportal.org/) [50].

## 5. Conclusions

The present case series study shows the first genotypic characterization of rs5945619, in a heterozygous distribution, in an Ecuadorian mestizo women cohort with BC. Given the small sample size and the absence of a control group, this distribution pattern cannot be interpreted as evidence of tumor-driven selective pressure; rather, it constitutes a preliminary observation that requires confirmation in population-based case–control studies with ancestry stratification.

In silico regulatory annotation positioned rs5945619 within a non-coding regulatory region and a high-confidence cis-regulatory element at Xp11.22. eQTL analyses suggested *NUDT11*, *GSPT2*, and *LINC01496* as potential transcriptional targets of this locus. However, the strongest regulatory evidence was derived mainly from normal prostate and testicular tissues, whereas GTEx showed only a modest breast-tissue eQTL signal for *GSPT2*. Thus, a tumor-specific regulatory effect in breast tissue cannot be inferred from the current data and requires validation through breast cancer-specific eQTL analyses and functional studies.

*NUDT11* expression has been documented in both normal and oncogenic breast tissue according to the Human Protein Atlas; however, this gene does not show prognostic significance in breast invasive carcinoma, and its functional role in BC biology has not been experimentally assessed. Moreover, although androgen receptor signaling is relevant in subsets of BC, the present data do not support direct long-range regulation of *AR* by rs5945619. Therefore, the underlying molecular mechanism of rs5945619 in BC pathogenesis remains to be determined and could be a field for future investigation.

In summary, this study identifies rs5945619 within the non-coding regulatory region as a candidate variant in BC pathogenesis in admixed Latin American populations. However, functional validation by breast cancer-specific eQTL analyses, histopathological studies, chromatin conformation assays in relevant breast tissue or tumor models, and larger case–control designs are warranted to establish the clinical and biological significance of this X-linked locus in BC susceptibility.

## Figures and Tables

**Table 1:** Clinical and pathological characteristics and rs5945619 T-allele frequencies in the study cohort and reference population.

Characteristics	Average ± SD
Age at diagnosis	54.5 ± 12.2 years
**Histopathological Type**	***N* (%)**
IBC-NST	17 (80.96%)
Invasive lobular	2 (9.52%)
Invasive mucinous carcinoma	1 (4.76%)
IBC-NST with micropapillary component	1 (4.76%)
**Immunohistochemical subtypes**	
Luminal A	11 (52.3%)
Luminal B	3 (14.3%)
HER2-enriched	3 (14.3%)
Triple-negative	3 (14.3%)
Unknown	1 (4.8%)
**rs5945619 detection**	
Heterozygous (C/T)	21 (100%)
Homozygous wild type (C/C)	0 (0%)
Homozygous variant (T/T)	0 (0%)
**Population (Source)**	**T-allele frequency**
Ecuadorian mestizo (Present study)	0.50
Latin American 1 (ALFA)	0.69
Latin American 2 (ALFA)	0.83
American (1000 Genomes)	0.82

IBC-NST: invasive breast carcinoma of no special type; ALFA: Allele Frequency Aggregator; Cohort frequency expressed as proportion of T alleles.

**Table 2:** Functional annotation summary.

Tool	Analysis	Result
RegulomeDB	Functional rank	1f
RegulomeDB	Functional score	0.22
GTEx (testis)	cis-eQTL: *LINC01496*	NES = +0.41 upregulation
GTEx (prostate)	cis-eQTL: *NUDT11* *LINC01496*	NES = −0.44 downregulation NES = −0.42 downregulation
GTEx (breast)	cis-eQTL: *GSPT2*	NES = −0.084 slight downregulation

eQTL: expression quantitative trait Locus; NES: normalized effect size.

## Data Availability

All data generated or analyzed during this study are included in this published article. Additional de-identified data are available from the corresponding author on reasonable request.
